# Prevalence of partial anomalous pulmonary venous connection in adult patients undergoing cardiac magnetic resonance for evaluation of atrial septal defect

**DOI:** 10.1186/1532-429X-15-S1-P285

**Published:** 2013-01-30

**Authors:** Venkateshwar Polsani, Faisal Nabi, Dipan J Shah

**Affiliations:** 1 Cardiology, DeBakey Heart and Vascular center, The Methodist Hospital, Houston, TX, USA

## Background

The association of partial anomalous pulmonary venous connection (PAPVC) with atrial septal defect (ASD) is known, but its prevalence in adult patients with ASD is not well reported in literature. CMR is recognized as excellent method for shunt quantification and assessment of ASD morphology.

## Methods

A total of 53 consecutive patients who were referred for evaluation of ASD by CMR from 2009-2011 were enrolled in the study. The patients with ASD in the setting of complex congenital heart disease were excluded. All patients were being considered for either percutaneous or surgical closure of ASD. The CMR protocol has been described previously and included standard SSFP (steady state free precision) cines to define the LV morphology and function, phase contrast was used to calculate the shunt fraction and define the ASD morphology, and a high resolution contrast enhanced 3-D MR angiograms were performed to assess pulmonary venous anatomy.

## Results

The total number of patients with ASD was 53. The baseline characteristics and pertinent CMR findings are shown in table 1. There were 45 (85%) patients with secundum ASD, 6 (11%) with primum ASD and 2 (4%) with sinus venosous ASD.

**Table 1:** Baseline demographics and the CMR findings

Patients(n)	55
Mean age	50.9 ±18.6

gender	34

Hypertension	18 (33%)

Hyperlipidemia	13(23%)

Diabetes mellitus	9 (16%)

ASD types	

Sinus Venosous	2 (3.7%)

Primum	6 (11.3%)

Secundum	45 (84.9%)

Mean LV ejection fraction	63.5±9.4

Mean RV ejection fraction	53± 10

Mean Qp: Qs	1.9±0.8

The prevalence of PAPVC in this population (n=53) was 6 (11%). The results of which are shown in the table 2. The PAPVC group included four patients with right superior pulmonary vein draining into the superior venacava, one right superior pulmonary vein draining into the right atrium and one left superior pulmonary vein draining into the left innominate vein.

**Table 2:** PREVALENCE OF PAPVC

ASD type	PAPVC
Total 9n=55)	8(14%)

Sinus Venosous (n=2)	1

Primum (n=6)	1

Secundum (n=45)	4

## Conclusions

The prevalence of PAPVC in adult congenital ASD patients referred for further evaluation of ASD is 14%. The incidence was higher in women (15%) compared to men (10%).

Since the presence of PAPVC will change the clinical decision of closure of ASD from percutaneous to surgical, the comprehensive CMR assessment for ASD can serve as an excellent non-invasive imaging modality not only to define the morphology and quantify shunt severity of ASD but also to identify the presence of PAPVC.

## Funding

None

